# Overexpression of miR-101 suppresses collagen synthesis by targeting EZH2 in hypertrophic scar fibroblasts

**DOI:** 10.1093/burnst/tkab038

**Published:** 2021-11-26

**Authors:** Jie Li, Yan Li, Yunchuan Wang, Xiang He, Jing Wang, Weixia Cai, Yanhui Jia, Dan Xiao, Jian Zhang, Ming Zhao, Kuo Shen, Zichao Li, Wenbin Jia, Kejia Wang, Yue Zhang, Linlin Su, Huayu Zhu, Dahai Hu

**Affiliations:** Department of Burns and Cutaneous Surgery, Xijing Hospital, Fourth Military Medical University, Xi'an, Shaanxi, 710032, China; Department of Burns and Cutaneous Surgery, Xijing Hospital, Fourth Military Medical University, Xi'an, Shaanxi, 710032, China; Department of Burns and Cutaneous Surgery, Xijing Hospital, Fourth Military Medical University, Xi'an, Shaanxi, 710032, China; Department of Burns and Cutaneous Surgery, Xijing Hospital, Fourth Military Medical University, Xi'an, Shaanxi, 710032, China; Department of Burns and Cutaneous Surgery, Xijing Hospital, Fourth Military Medical University, Xi'an, Shaanxi, 710032, China; Department of Burns and Cutaneous Surgery, Xijing Hospital, Fourth Military Medical University, Xi'an, Shaanxi, 710032, China; Department of Burns and Cutaneous Surgery, Xijing Hospital, Fourth Military Medical University, Xi'an, Shaanxi, 710032, China; Department of Burns and Cutaneous Surgery, Xijing Hospital, Fourth Military Medical University, Xi'an, Shaanxi, 710032, China; Department of Burns and Cutaneous Surgery, Xijing Hospital, Fourth Military Medical University, Xi'an, Shaanxi, 710032, China; Department of Burns and Cutaneous Surgery, Xijing Hospital, Fourth Military Medical University, Xi'an, Shaanxi, 710032, China; Department of Burns and Cutaneous Surgery, Xijing Hospital, Fourth Military Medical University, Xi'an, Shaanxi, 710032, China; Department of Burns and Cutaneous Surgery, Xijing Hospital, Fourth Military Medical University, Xi'an, Shaanxi, 710032, China; Department of Burns and Cutaneous Surgery, Xijing Hospital, Fourth Military Medical University, Xi'an, Shaanxi, 710032, China; Department of Burns and Cutaneous Surgery, Xijing Hospital, Fourth Military Medical University, Xi'an, Shaanxi, 710032, China; Department of Burns and Cutaneous Surgery, Xijing Hospital, Fourth Military Medical University, Xi'an, Shaanxi, 710032, China; Department of Burns and Cutaneous Surgery, Xijing Hospital, Fourth Military Medical University, Xi'an, Shaanxi, 710032, China; Department of Burns and Cutaneous Surgery, Xijing Hospital, Fourth Military Medical University, Xi'an, Shaanxi, 710032, China; Department of Burns and Cutaneous Surgery, Xijing Hospital, Fourth Military Medical University, Xi'an, Shaanxi, 710032, China

**Keywords:** Hypertrophic scars, miR-101, EZH2, Collagen, Fibroblasts, Skin, Collagen

## Abstract

**Background:**

MicroRNA-101 (miR-101) is a tumor suppressor microRNA (miRNA) and its loss is associated with the occurrence and progression of various diseases. However, the biological function and target of miR-101 in the pathogenesis of hypertrophic scars (HS) remains unknown.

**Methods:**

We harvested HS and paired normal skin (NS) tissue samples from patients and cultured their fibroblasts (HSF and NSF, respectively). We used quantitative reverse transcriptase polymerase chain reaction (qRT-PCR), fluorescence *in situ* hybridization (FISH), enzyme-linked immunosorbent assays (ELISA) and Western blot analyses to measure mRNA levels and protein expression of miR-101, enhancer of zeste homolog 2 (EZH2), collagen 1 and 3 (Col1 and Col3) and α-smooth muscle actin (α-SMA) in different *in vitro* conditions. We also used RNA sequencing to evaluate the relevant signaling pathways and bioinformatics analysis and dual-luciferase reporter assays to predict miR-101 targets. We utilized a bleomycin-induced fibrosis mouse model in which we injected miR-101 mimics to evaluate collagen deposition *in vivo*.

**Results:**

We found low expression of miR-101 in HS and HSF compared to NS and NSF. Overexpressing miR-101 decreased Col1, Col3 and α-SMA expression in HSF. We detected high expression of EZH2 in HS and HSF. Knockdown of EZH2 decreased Col1, Col3 and α-SMA in HSF. Mechanistically, miR-101 targeted the 3′-untranslated region (3′UTR) of EZH2, as indicated by the decreased expression of EZH2. Overexpressing EZH2 rescued miR-101-induced collagen repression. MiR-101 mimics effectively suppressed collagen deposition in the bleomycin-induced fibrosis mouse model.

**Conclusions:**

Our data reveal that miR-101 targets EZH2 in HS collagen production, providing new insight into the pathological mechanisms underlying HS formation.

Highlights

- This study is the first to examine miR-101 biological function in hypertrophic scar and its target gene EZH2.
- This study is the first to show that knockdown of EZH2 represses hypertrophic scar fibroblasts collagen synthesis.

## Background

Hypertrophic scars (HS) are an intractable complication associated with abnormal wound healing after skin injury. HS are characterized by the excessive proliferation of fibroblasts and excessive deposition of extracellular matrix (ECM) proteins [1]. In these processes, hypertrophic scar fibroblasts (HSF) exhibit abnormal features, including excessive deposition and alterations in collagen morphology. However, the molecular mechanism underlying HS formation remains largely unclear [2,3].

Enhancer of zeste homolog 2 (EZH2) is a histone-lysine *N*-methyltransferase that has been shown to function as an oncogene in tumorigenesis, participating in histone methylation and transcriptional repression [4]. Overexpression of EZH2 is a predictor of poor clinical outcomes in breast cancer patients and is associated with breast cancer metastasis [5]. Destabilizing EZH2 has been shown to induce cell cycle arrest and inhibit prostate cancer progression [6]. However, the effects of EZH2 overexpression on HS formation remain unknown. Moreover, there is no evidence on the effects of EZH2 knockdown as a therapeutic intervention for HS.

Emerging evidence suggests that mircoRNA-101 (miR-101) acts as a tumor suppressor microRNA (miRNA) in tumorigenesis. Recent studies have revealed that overexpression of miR-101 can decrease cell proliferation, migration and invasion, as well as induce apoptosis and irradiation sensitivity [7–10]. Alterations of miR-101 underlie various biological events in solid tumors, but the expression and biological role of miR-101 have not been shown in HSF. Comparisons of miR-101 expression in normal skin and HS could identify possible mRNA targets underlying formation of HS and therefore provide molecule targets for therapeutic strategies or prevention. In this study, we investigated the expression of miR-101 and EZH2 in HS and elucidated the role of EZH2 in collagen synthesis. We demonstrated that miR-101 positively regulates EZH2 expression by binding to its 3′-untranslated region (3′UTR) leading to inhibition of EZH2 translation. MiR-101 also reduced bleomycin-induced fibrosis in our mouse model, providing new insight into the underlying molecular mechanisms and therapeutic strategies of oncogenic miRNAs in fibrotic diseases.

## Methods

### HS tissue samples and cell culture

HS tissues samples and paired normal skin (NS) tissue samples were obtained from nine patients at the Xijing Hospital (Xi’an, China). Informed consent was obtained from each patient and all protocols were approved by the Ethics Committee of Xijing Hospital affiliated with the Fourth Military Medical University (register number: KY20173012-1). Diagnosis was confirmed by routine pathological examination. All HS patient details are listed in Table 1. The tissues were digested with 0.25% collagenase type I (Sigma, Germany) at 37°C for 0.5 h. The digested cells were filtered through a 75 μm cell filter and then cultured in Dulbecco’s modified Eagle medium (DMEM; Gibco, USA) containing 10% fetal bovine serum (FBS; Gibco) at 5% CO_2_ and 37°C. Primary HSF and normal skin fibroblasts (NSF) from the third culture generation were used. HEK293T and HEK293A cells were cultured in RPMI 1640 at 5% CO_2_ and 37°C [11] for lentivirus packaging and luciferase assays, respectively.

**Table 1:** Tissue sample data used in this study

**Sample number**	**Gender**	**Age (years)**	**Site**
H1,N1	Female	47	Abdomen
H2,N2	Male	35	Arm
H3,N3	Female	3	Abdomen
H4,N4	Female	36	Abdomen
H5,N5	Male	38	Abdomen
H6,N6	Male	38	Arm
H7,N7	Male	35	Arm
H8,N8	Male	25	Abdomen
H9,N9	Male	7	Abdomen

### Cell transfection of miR-101 siRNA, mimics and inhibitor

HSF were grown in 6-well plates and the synthesized RNAs (100 mM of diluted siRNA, miR-101 mimics or miR-101 inhibitor per well) were transfected using Lipofectamine 2000 (Invitrogen, Carlsbad, CA, USA). After 24 or 48 h transfection, the cells were harvested for analysis. Carboxy Fluorescein (FAM) modified 2′-OMe-oligonucleotides were chemically synthesized and purified using high-performance liquid chromatography (GenePharma, Shanghai, China). All transfections were performed in triplicate. The primer sequences were as follows: siRNA-EZH2: 5′-UAGCUUAUCAGACUGAUGUUGA-3′; siRNA-control: 5′-UAGCUUAUCAGACUGAUGUUGA-3′; miR-101 mimics: 5′-CUACAGUACUGUGAUAACUGAA-3′, mimics control: 5′-UAGCUUAUCAGACUGAUGUUGA-3′, miR-101 inhibitor: 5′-GAUGUCAUGACACUAUUGACUU-3′, miR-101 inhibitor control: 5′-CAGUACUUUUGUGUAGUACAA-3′.

### RNA sequencing and bioinformatics analysis

RNA sequencing was used to analyze the gene expression profiles of HSF transfected with siRNA-EZH2 and siRNA-control. Total RNA from HSF was extracted using the RNeasy Mini Kit (QIAGEN, Düsseldorf, Germany). After RNA extraction, the enriched mRNA was fragmented into short fragments and reverse-transcribed into cDNA with random primers. The cDNA fragments were purified and ligated to Illumina sequencing adapters. The ligation products were sequenced on an Illumina HiSeq2500 by Gene Denovo Biotechnology Co. (Guangzhou, China). All raw transcriptome data have been deposited in the Sequence Read Archive (SRA) in the NCBI (accession numbers PRJNA729564). Gene expression fold change of <0.5 was used for cluster-based analysis of the biological processes dysregulated following EZH2 knockdown using the ClueGO + CluePedia tool in Cytoscape [12]. Gene ontology was performed using the DAVID Bioinformatic Resources (https://david.ncifcrf.gov/) to identify categories of functional pathways [13]. The binding sites of miR-101 and EZH2 3′UTR were predicted using the TargetScan database (http://www.targetscan.org/mamm_31/).

### RNA extraction and quantitative real-time polymerase chain reaction assay

For the mRNA quantitative real-time polymerase chain reaction (qRT-PCR) assay, total cell RNA was extracted using Trizol (Takara, RNAiso Plus Kit). The 2^−ΔΔCT^ method was used to determine relative gene expression. For the miRNA qRT-PCR assay, RNA was reverse-transcribed into cDNA using an Mir-X™ First Strand Synthesis Kit (Takara). qRT-PCR was performed in triplicate on a Bio-Rad IQ5 Real-Time system (Bio-Rad, Hercules, CA, USA). Real-time PCR was performed using an SYBR® Premix Ex-Taq™ Kit (Takara, Bio Inc., Otsu, Japan). Glyceraldehyde-3-Phosphate Dehydrogenase (GAPDH) and U6 RNA were used as internal loading controls for the mRNA and miRNA qRT-PCR assays. The primer pairs were as follows: Collagen type I (Col1): forward 5′-GAG GGC AAC AGC AGG TTC ACTTA-3′ and reverse 5′-TCA GCA CCA CCG ATG TCC A-3′; collagen type III (Col3): forward 5′-CCA CGG AAACAC TGG TGG AC-3′ and reverse 5′-GCC AGC TGC ACATCA AGG AC-3′; α-smooth muscle actin (α-SMA): forward 5′-GAC AAT GGC TCTGGG CTC TGTAA-3′ and reverse 5′-TGT GCT TCG TCA CCC ACG TA-3′; EZH2: forward 5’-ACA TGC GAC TGA GAC AGC TCA AG −3′ and reverse 5′-TCC AAG TCA CTG GTC ACC GAA CA-3′; GAPDH: forward 5’-GCA CCG TCA AGC TGA GAA C-3′ and reverse 5′-TGG TGA AGA CGC CAG TGG A-3′; miR-101 forward 5′-CTA CAG TAC TGT GAT AAC TGA A − 3′ U6 forward 5′-GTG CTC GCT TCG GCA GCA CAT AT-3′; Universal Primer (QIAGEN) was used as reverse primer.

### Western blot analysis

To obtain total protein, tissues or HSF were lysed in RIPA buffer (Heart Biological Technology Co. Ltd, China) with Phenylmethanesulfonyl Fluoride (PMSF) added on ice. For western blot analysis, 30 μg of total protein was loaded onto a 10% Dodecyl Sulfate, Sodium Salt (SDS)-Polyacrylamide Gel Electrophoresis (SDS-PAGE) gel, separated and transferred to a Polyvinylidene Difluoride (PVDF) membrane at 100 V for 60–80 min. The membranes were blocked with 5% nonfat milk and then incubated with primary antibodies at 4°C overnight. The next day, the membrane was washed and incubated with secondary antibodies (1:3000; Cell Signaling Technology (CST)) at 37°C for 2 h. Immunoreactive proteins were then visualized using a chemiluminescence system with an Enhanced Chemiluminescence (ECL) reagents kit (Millipore, USA). Protein band intensity was detected using the Fluor Chem FC system (Alpha Innotech, USA). Antibodies were as follows: anti-Col1 (rabbit, 1:1000; Abcam), anti-Col3 (rabbit, 1:3000; Abcam), anti-α-SMA (rabbit, 1:1000; Abcam), anti-EZH2 (rabbit, 1:1000; Abcam) and anti-GAPDH (Rabbit, 1:3000; Abcam).

### Plasmid construction and lentiviral packaging

EZH2 plasmids containing the open reading frame (ORF) region sequence were generated by GeneCopoeia (Shanghai, China). Plasmids containing the wild-type and mutation EZH2 3′UTR sequence were cloned into the pGL3 luciferase vector target (Promega, WI, USA) with forward primer, 5′-ATA AAT ACA TGT GCA GCT TT-3′, and reverse primer, 5′-GAC AAG TTC AAG TAT TCT T-3′. The PCR products to generate the mutation EZH2 3′UTR Mut-EZH2-3′UTR vector included forward primer, 5′-CTT CAG GAA CCT CGA ATG ACA TG-3′ and reverse primer, 5′-TTT CTA AAT TGC CCA TGT CAT GTC G-3′. The constructs were sequence verified. EZH2 plasmids containing the ORF were packaged into the lentivirus packaging plasmid pLenti 6.3 (GeneChem Co., Ltd, Shanghai, China) and then transfected into HEK293T cells for 48 h. The infected HSF were cultured in DMEM containing 10% FBS and collected after 72 h post-infection for analysis.

### Dual-luciferase reporter assay

HEK-293A cells at 50% to ~90% confluence were added to 48-well plates and transfected with miR-101 mimics or control (20 pmol) along with the firefly luciferase reporter gene construct (EZH2 3′UTR and Mut-EZH2-3′UTR, 100 ng) using Lipofectamine 2000 (Invitrogen, Carlsbad, CA, USA). Renilla luciferase construct (5 ng) was co-transfected for normalization with the dual-glo luciferase assay system (Promega, Madison, WI, USA). After 48 h, cells were lysed and the luciferase activity was measured using the luciferase reporter assay system (Promega).

### Bleomycin-induced fibrosis mouse model

All animal studies were approved and carried out in strict accordance with the Institutional Animal Care policies. Five-week-old male BALB/c mice were purchased from the Fourth Military Medical University Laboratory Animal Center (Xian, China) and housed under standard conditions. Mice were randomly divided into three groups (*n* = 6 per group): phosphate-buffered saline (PBS) control group, bleomycin (500 μg/mL) group and bleomycin (500 μg/mL) plus miR-101 mimics group. All mice were shaved and injected subcutaneously in the backs to establish the HS mouse model [14,15]. Mice were sacrificed after 4 weeks and lesioned skin tissues were subjected to hematoxylin and eosin (H&E) and Masson’s trichrome staining analyses.

### Immunofluorescence, H&E and Masson’s trichrome staining

For HS and NS immunohistological staining, paraffin-embedded tissue sections were blocked with 1% bovine serum albumin (BSA) for 1 h at room temperature to inhibit nonspecific binding of IgG. Tissue sections were then incubated with rabbit anti-human EZH2 antibody (1:500; Abcam) overnight at 4°C, followed by incubation with anti-rabbit Cy3 IgG for 1 h at 37°C. Nuclei were stained with 4′, 6-Diamidino-2′-phenylindole (DAPI). Sections were counterstained with hematoxylin to examine pathological fibrogenesis. For Masson’s trichrome staining, the tissue sections were deparaffinized with graded ethanol together with xylene, then stained with Masson’s trichrome to examine collagen fibers. Images were taken with an FSX100 fluorescence microscope (Olympus, Tokyo, Japan).

### RNA fluorescence *in situ* hybridization

The miR-101 probe (5′-FAM- TGT CTA TTC TAA AGG TAC AGT AC-FAM-3′) was applied for RNA fluorescence *in situ* hybridization (FISH). First, the probe was marked with DIG-UTP (Roche) for RNA labeling. Cell climbing was fixed in 4% paraformaldehyde ( Diethyl Pyrocarbonate) for 20 min [16]. We added proteinase K (20 μg/ml) and incubated at 37°C for 5 min and then incubated the probe at 37°C for 1 h followed by incubation in the probe hybridization solution at 4°C overnight. We washed the slides successively in 2 × Saline Sodium Citrate buffer (SSC), 1 × SSC and 0.5 × SSC. The slides were then incubated with DAPI for 8 min in the dark and imaged using light microscopy.

### Enzyme-linked immunosorbent assay

Lysed cells were subjected to an enzyme-linked immunosorbent assay (ELISA) for Collagen Type I (Cloud-clone, SEA571Hu) and Collagen Type III (Cloud-clone, SEA176Hu) according to the manufacturer’s kit instructions.

### Statistical analysis

All measurement data are expressed as the mean ± standard deviations (SD) of at least three separate experiments unless otherwise indicated. Statistical analysis of all data was performed using Prism version 11 (GraphPad Software, CA, USA) statistical software packages. Data were analyzed as follows: (1) two-tailed Student t test for *in vitro* cell line experiments and *in vivo* analysis, including luciferase assay, qRT-PCR, ELISA, cell growth assay, dermal thickness and collagen content analysis; (2) Wilcoxon nonparametric test for paired data in gene expression analysis; and (3) significance of associations between miR-101 and EZH2 expression values was determined using Pearson’s product–moment correlation coefficient. Differences were considered significant when *p* < 0.05 (^*^), *p* < 0.01 (^*^^*^) or *p* < 0.001 (^*^^*^^*^).

## Results

### miR-101 is down-regulated in HS and HSF

**Figure 1.**
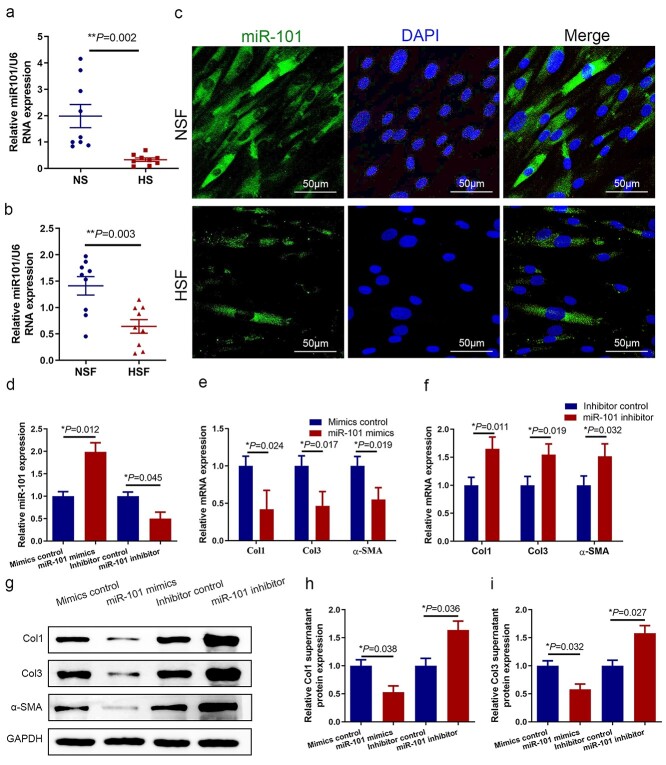
Overexpression of miR-101 decreased fibrosis-related protein expression and proliferation. miR-101 RNA levels were analyzed using qRT-PCR normalized to U6 in HS (**a**) and HSF (**b**), compared with NS and NSF. Each data point was normalized against its corresponding U6 level. (**c**) FISH staining for miR-101 using serial sections of clinical HS and paired NS. Scale bar = 50 μm. (**d**) qRT-PCR analysis of the effects of mimics control, miR-101 mimics, inhibitor control and miR-101 inhibitor on the level of miR-101. qRT-PCR analysis (**e**, **f**) and representative immunoblots (**g**) showing the mRNA level and protein level changes of Col1, Col3 and α-SMA in HSF transfected with mimics control, miR-101 mimics, inhibitor control and miR-101 inhibitor. (**h**, **i**) Col1 and Col3 supernatant protein levels were determined using ELISA in the same treatment. Data are expressed as the mean ± SD of three independent experiments. ^*^p < 0.05, ^*^^*^p < 0.01. *qRT-PCR* quantitative reverse-transcriptase polymerase chain reaction, *U6* U6 small nuclear 1, *HS* hypertrophic scar, *HSF* hypertrophic scar fibroblast, *NS* normal skin, *NSF* normal skin fibroblast, *FISH* fluorescence *in situ* hybridization, *ELISA* enzyme-linked immunosorbent assay.

To confirm the potential function of miR-101 in HS, we measured miR-101 expression levels in nine pairs of HS and NS tissue samples (Table 1), as well as in their derived fibroblasts (NSF and HSF). qRT-PCR analysis showed that miR-101 expression was significantly decreased in HS compared to NS tissues (Figure 1a), and the same trend was observed in the fibroblast analysis (Figure 1b). We utilized FISH to study miR-101 localization and expression levels in NSF and HSF, and found that the expression of miR-101 was lower in HSF compared to NSF (Figure 1c). The green fluorescent distribution indicated that miR-101 was mainly localized in the cytoplasm of fibroblasts. These results indicate that miR-101 expression is related to HS formation.

Since miR-101 has been shown to act as a tumor suppressor, we hypothesized that miR-101 could regulate collagen deposition of fibroblasts in HS. HSF were transfected with mimics to increase or an inhibitor to attenuate miR-101 expression (Figure 1d). The major characteristics of fibrosis are excessive abnormal deposition of collagen, mainly Col1 and Col3. qRT-PCR analysis showed that the expression levels of Col1, Col3 and α-SMA were significantly decreased in the cells transfected with miR-101 mimics compared to the cells transfected with control mimics, whereas the miR-101 inhibitor down-regulated expression of these molecules (Figure 1e, f). These findings were confirmed using western blot analysis (Figure 1g). We also assessed the supernatant protein levels of Col1 and Col3 using ELISA and found that miR-101 expression promoted anti-fibrotic effects in HS. These results demonstrate that miR-101 is an important regulator of fibrosis-related protein expression in HS.

### EZH2 expression is up-regulated in patients with HS

**Figure 2.**
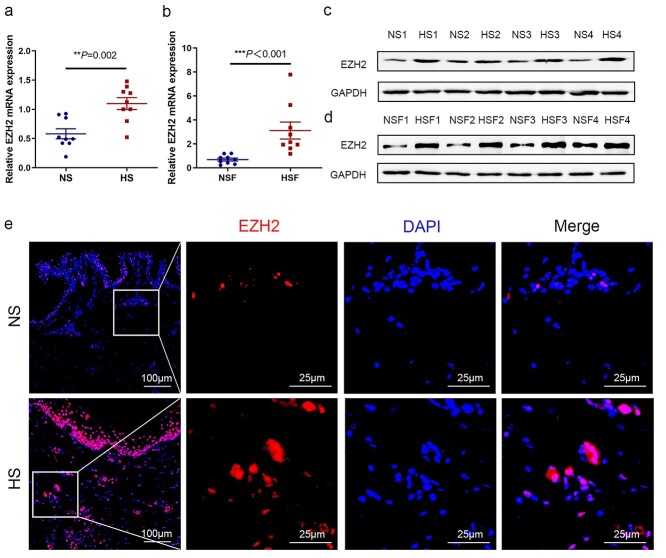
Up-regulation of EZH2 in HS and HSF. EZH2 mRNA levels were determined using qRT-PCR in NS and HS tissues (**a**) or in NSF and HSF (**b**). In the same samples, representative western blot analysis of EZH2 expression in NS and HS tissues (**c**) or in NSF and HSF (**d**). (**e**) Immunofluorescence analysis of EZH2 protein in HS and paired NS tissues; the image on the right is an enlarged image of the box on the left. Scale bar = 100 μm (left) and 25 μm (right). Data are expressed as the mean ± standard deviation (SD). ^*^^*^p < 0.01, ^*^^*^^*^p < 0.001. *EZH2* enhancer of zeste homolog 2, *qRT-PCR* quantitative reverse-transcriptase polymerase chain reaction, *NS* normal skin, *HS* hypertrophic scar, *NSF* normal skin fibroblast, *HSF* hypertrophic scar fibroblast.

EZH2 is an oncogene that is broadly overexpressed in solid tumors. However, the biological role of EZH2 in HS remains unknown. We first examined EZH2 expression in HS and HSF. We observed that EZH2 mRNA levels and protein expression were highly overexpressed in HS and HSF compared to NS and NSF (Figure 2a–d). EZH2 expression was up-regulated in HS compared to NS tissue samples as determined by immunofluorescence staining (Figure 2e), suggesting that EZH2 overexpression is prominent in patients with HS.

### Knockdown of EZH2 decreases fibrosis-related protein expression

**Figure 3.**
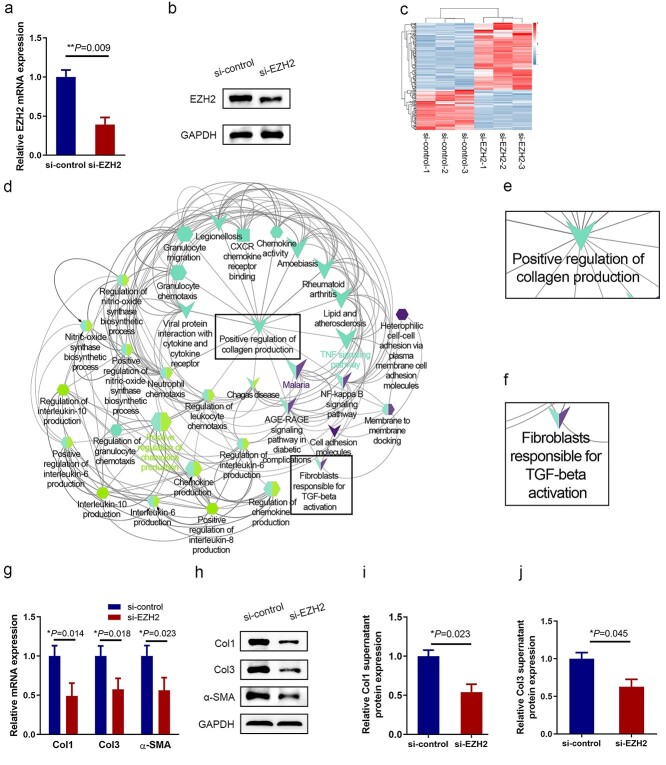
The role of EZH2 in regulating collagen and α-SMA expression in HSF. EZH2 mRNA (**a**) and protein expression (**b**) were measured using qRT-PCR and western blot analyses, respectively, in siRNA-EZH2 and siRNA-control transfected HSF. (**c**) RNA sequencing profiling of HSF in which EZH2 was knocked down. Shades of red represent increased gene expression; shades of blue represent decreased gene expression. (**d**) Pathway enrichment was done using the ClueGO and CluePedia plugins of the Cytoscape software. This analysis identified seven enriched pathways based on statistical analysis, with no duplication between clusters. Cluster one mainly includes positive regulation of collagen production (**e**) and fibroblasts responsible for TGF-β activation (**f**). Immunoblotting (**g**) or qRT-PCR (**h**) assessing the effect of EZH2-siRNA on the expression of Col1, Col3 and α-SMA in HSF. (**i**, **j**) ELISA of Col1 and Col3 supernatant protein levels in EZH2 knocked down HSF. *EZH2* enhancer of zeste homolog 2, *qRT-PCR* quantitative reverse-transcriptase polymerase chain reaction, *HSF* hypertrophic scar fibroblast, *ELISA* enzyme-linked immunosorbent assay

To detect the biological function of EZH2 in HS, EZH2 was knocked down using siRNA (Figure 3a, b). We profiled differentially expressed genes and pathway changes induced by EZH2 knockdown using RNA sequencing analysis (Figure 3c). Cytoscape analysis indicated that most EZH2-regulated genes were enriched in positive regulation of collagen production and fibroblasts responsible for transforming growth factor beta (TGF-β) activation (Figure 3d–f). We also found that EZH2 expression positively correlated with collagen deposition (Figure 3g–j). Results showed that EZH2 siRNA suppressed the synthesis of collagen and expression of α-SMA at both the mRNA and protein levels, paralleling the effects of the miR-101 mimics.

### EZH2 is a direct target of miR-101

**Figure 4.**
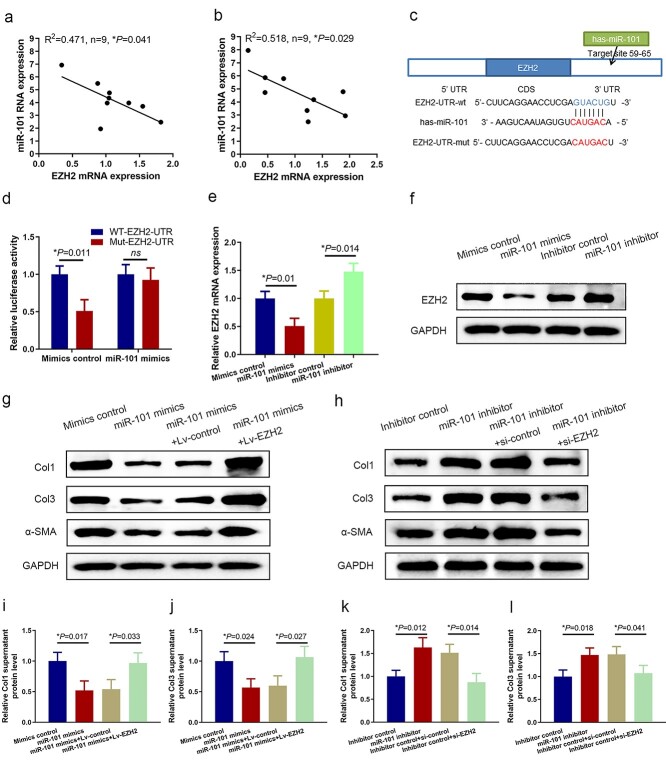
miR-101 targets EZH2 in HSF. Expression levels of miR-101 are negatively correlated with EZH2 expression in HS (**a**) and HSF (**b**). Pearson’s correlation analysis of the relative expression levels of miR-101 (normalized to U6) and EZH2 (normalized to GAPDH) was determined using qRT-PCR in nine paired HS and HSF samples. (**c**) Binding of miR-101 to the wild type EZH2 3′ UTR (WT-EZH2-UTR) and the designed mutant (Mut-EZH2-UTR). (**d**) Dual-luciferase reporter assay showing that miR-101 mimics reduced luciferase activity in HEK293A cells after transfection with WT- EZH2-UTR, but not with the Mut- EZH2-UTR vector. Analysis of expression levels of EZH2 in HSF infection with miR-101 and miR-101 inhibitor using qRT-PCR (**e**) and western blot (**f**) analyses. Western blot analysis of Col1, Col3 and α-SMA protein expression in HSF treated with miR-101 mimics or infected with lentivirus overexpressing EZH2. (**h**) Western blot analysis of Col1, Col3 and α-SMA protein expression in HSF treated with miR-101 inhibitor or EZH2 siRNA. ELISA analysis of Col1 (**i**) and Col3 (**j**) supernatant protein level treated with miR-101 mimics or infected with lentivirus overexpressing EZH2. ELISA analysis of Col1 (**k**) and Col3 (**l**) supernatant protein level treated with miR-101 inhibitor or EZH2 siRNA. *EZH2* enhancer of zeste homolog 2, *HS* hypertrophic scar, *HSF* hypertrophic scar fibroblast, *U6* U6 small nuclear 1, *UTR* untraslated region, *WT* wild type, *Mut* mutant, *HEK293A* human embryonic kidney 293A cell, *ELISA* enzyme-linked immunosorbent assay

Based on our observation that both miR-101 and EZH2 can regulate collagen deposition and α-SMA expression, we postulated that miR-101 expression may be correlated with EZH2 expression in human HS. To test this hypothesis, we measured miR-101 and EZH2 levels in paired HS and NS tissues, as well as in their paired HSF and NSF, using qRT-PCR. We found that miR-101 levels were negatively correlated with EZH2 mRNA levels (Figure 4a, b). To explore the mechanism, we predicted potential EZH2 targets using Target scan and found that the EZH2 3′UTR was a potential miR-101 target gene (Figure 4c). EZH2-UTR or EZH2-UTR-mutant (mut) reporter plasmids were co-transfected into HSF along with the miR-101 mimics or scrambled control. The relative luciferase activity of EZH2-UTR was significantly repressed by miR-101 transfection. However, the mutant reporter plasmid abolished the miR-101 mediated decrease in luciferase activity (Figure 4d). Consistently, overexpression of miR-101 in HSF downregulated EZH2 protein expression and miR-101 levels (Figure 4e, f). To test whether overexpression of EZH2 could antagonize the impact of miR-101 on Col1, Col3 and α-SMA, we transfected exogenous EZH2 into miR-101-overexpressing HSF. We observed an increase in collagen and α-SMA expression (Figure 4g). Knockdown of EZH2 using siRNA in miR-101-deregulated HSF significantly decreased collagen and α-SMA expression, indicating that EZH2 is a functional mediator of miR-101 in HS (Figure 4h).

**Figure 5.**
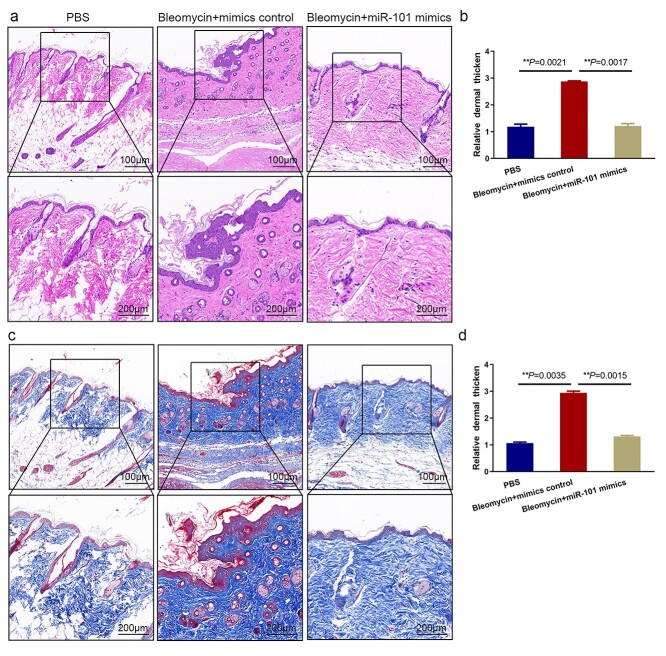
Bleomycin-induced dermal fibrosis is inhibited by miR-101. The thickness (**a**) and analysis (**b**) of the dermal layer in PBS, bleomycin and bleomycin plus miR-101 mimics-treated mice were measured using H&E staining. Scale bar = 200, 100 μm (*n* = 6 mice per group). Masson’s trichrome staining (**c**) and analysis (**d**) in the same treatments. Scale bar = 200, 100 μm (*n* = 6 mice per group). *PBS* phosphate buffer saline, *H&E* hematoxylin & eosin

### MiR-101 mimics attenuate bleomycin-induced fibrosis in BALB/c mice

To determine if miR-101 promotes collagen deposition and expression *in vivo*, we established the bleomycin-induced fibrosis mouse model. We injected miR-101 mimics after 2 weeks of subcutaneous bleomycin injections. H&E staining demonstrated that the dermal layer of the mouse back became thinner after injection of miR-101 mimics compared to the bleomycin-induced and PBS groups (Figure 5a, b). Masson’s trichrome staining demonstrated an ordered arrangement of collagen in the miR-101 mimics group, while there was excessive and disordered collagen deposition in the bleomycin-induced group (Figure 5c, d). These results suggest that miR-101 mimics attenuate bleomycin-induced fibrosis *in vivo*.

## Discussion

The most important finding from this study is that EZH2-induced collagen deposition can be repressed by miR-101 in HS. miR-101 overexpression in HSF showed significantly reduced collagen production in the control groups. Consistently, we observed a noticeable reduction in collagen production following EZH2 knockdown in HSF. Mechanistically, bioinformatics analysis showed that miR-101 binds the EZH2 3′UTR and directly reduces EZH2 expression, indicating that miR-101 overexpression could be used in anti-fibrosis clinical application.

After skin injury, fibroblasts around the wound rapidly proliferate and secrete ECM, which is mainly composed of collagen I/III, to promote wound healing. These fibroblasts can transdifferentiate into myofibroblasts (MFB) in response to a variety of cytokines, mechanical tension, and ECM. Under pathological conditions, inflammatory factors, cytokines, mechanical forces and other factors induce the vigorous proliferative capacity of MFB and the excessive deposition of ECM proteins, thereby forming HS [17,18]. It has been reported that ~40%–70% of surgical damage and >91% of burn injuries result in HS. Unfortunately, the underlying mechanisms involved in HS and how to alter collagen deposition in the ECM remain to be elucidated.

Emerging evidence has demonstrated an important role of miRNA in scar pathogenesis. MiRNAs are short, non-coding RNAs of ~18–22 nucleotides that can regulate gene expression by binding to the 3′-UTR of specific mRNAs to regulate post-transcriptional gene expression and block translation or degradation. Bioinformatics analyses have indicated that >50% of all genes are regulated by miRNAs [19,20]. Recent studies have shown that abnormal miRNA expression is associated with fibrotic diseases, such as lung fibrosis, hepatic fibrosis, cardiac fibrosis and systemic sclerosis skin fibrosis. Our findings presented here indicate that miR-101 is also involved in HS fibrosis.

MiR-101 has been shown to function as a tumor suppressor miRNA, and it is down-regulated in many tumors because of genomic loss [21–25]. Several studies have shown that miR-101 regulates fibrotic processes, such as collagen deposition and α-SMA gene expression [26–28]. Zhao *et al.*, found that miR-101 overexpression decreased collagen and α-SMA expression, indicating that miR-101 is a potential target in acute kidney injury to chronic kidney disease transition [29]. MiR-101 has also been shown to significantly inhibit hepatic stellate cell LX-2 proliferation and reduce high accumulation of ECM induced by TGF-β1, as well as negatively control collagen deposition and α-SMA expression [30]. Overexpression of miR-101a/b suppressed collagen production and cell proliferation in rat neonatal cardiac fibroblasts, suggesting the therapeutic potential of miR-101a in cardiac disease associated with fibrosis [31]. Results from our *in vitro* functional studies provide evidence that miR-101 directly regulates collagen deposition in HFS, and we confirmed *in vivo* that miR-101 can directly inhibit collagen deposition in bleomycin-induced fibrosis. Our results further confirmed that that EZH2 is a direct target of miR-101, indicating that miR-101 could be explored as an anti-fibrotic therapeutic in pre-clinical applications.

EZH2 has been reported to be dysregulated at genetic, transcriptional and post-transcriptional levels in carcinomas. Its overexpression has also been associated with poor prognosis in several cancers [32]. This suggests that EZH2 is not only a therapeutic target, but also a potential tumor biomarker. In this study, we demonstrated that EZH2 was highly expressed in HS and fibroblasts, and its expression levels were positively correlated with collagen I/III and α-SMA expression.

The literature demonstrates that EZH2 plays an important role in regulating fibrosis. Jiang *et al*., found that EZH2 inhibition resulted in decreased hepatic collagen deposition, especially down-regulation of cellular and hepatic Col1A and α-SMA expression, providing new mechanistic insight into hepatic fibrogenesis [33]. Xu *et al*. [34] and Lim *et al*. [35] found that down-regulation of EZH2 correlated with reduced extracellular collagen deposition in renal tubulointerstitial fibrosis and liver fibrosis. Shi *et al*. found that siRNA-mediated knockdown of EZH2 enhanced TGF-β1-induced up-regulation of collagen and α-SMA in peritoneal fibrosis [36]. Moreover, our genome-wide data confirm that knockdown of EZH2 directly inhibits collagen synthesis in HSF. Based on the literature and our findings presented here, EZH2 is an attractive target molecule in HS. However, the direct target of EZH2 in HS remains unclear. Our Kyoto Encyclopedia of Genes and Genomes (KEGG) pathway analysis revealed that EZH2-regulated genes were enriched in tumor necrosis factor and interleukin-17 signaling pathways. Thus, we propose that EZH2 activates the inflammatory response during fibrogenesis, but further investigation is needed to test this hypothesis. Future studies will focus on the downstream target of EZH2 using techniques such as co-immunoprecipitation to investigate the possible interactions between EZH2 and inflammatory factors in HS.

## Conclusions

Our study confirmed that miR-101 loss occurs in HS and that knockdown of EZH2 represses HSF collagen synthesis. We explored the possibility that miR-101 targets EZH2 gene expression through preventing collagen expression, providing new insights into the mechanism of HS formation and novel potential anti-skin fibrosis therapeutic targets.

**Abbreviations**
Col I: Collagen I; Col III: Collagen III; DMEM: Dulbecco’s modified Eagle medium; ECL: enhanced chemiluminescence; ECM: Extracellular matrix; ELISA: Enzyme-linked immunosorbent assay; EZH2: Enhancer of zeste homolog 2; FBS: Fetal bovine serum; H&E: Hematoxylin and eosin; HS: Hypertrophic scar; HSF: Hypertrophic scar fibroblasts; MFB: Myofibroblasts; miRNA: MicroRNA; NS: Normal skin; NSF: Normal skin fibroblasts; ORF: Open reading frame; PBS: Phosphate-buffered saline; qRT-PCR: Quantitative reverse transcriptase polymerase chain reaction; 3′-UTR: 3′-Untranslated region; α-SMA:
  α-Smooth muscle actin; TGF- β1: Transforming growth factor-β1; 3′-UTR: 3′-Untranslated region.

## Authors’ contributions

JL, YL and YCW performed the study, analyzed the data, drafted the manuscript and approved the final manuscript. XH, JW, WXC, YHJ and DX were involved in conception and design, collection and/or assembly of data, performed experiments and data analysis. JZ, MZ, KS and ZCL analyzed and interpreted data from experiments. WBJ, KJW and YZ revised the manuscript. DHH, HYZ and LLS contributed to the conceptual design of the study, interpreted the data, provided funding support and critically revised the manuscript. All authors have given final approval and agree to be accountable for all aspects of the work.

## Funding

This study was supported by the National Natural Science Foundation of China (81772071 to DHH).

## Availability of data and materials

The datasets used and/or analyzed in the current study are available from the corresponding author upon reasonable request.

## Ethics approval and consent to participate

This study was approved by the Ethics Committee of Xijing Hospital affiliated to the Fourth Military Medical University.

## Competing interests

None declared.
